# Determination of Patulin in Apple Juice and Apple-Derived Products Using a Robotic Sample Preparation System and LC-APCI-MS/MS

**DOI:** 10.3390/toxins16060238

**Published:** 2024-05-23

**Authors:** Kai Zhang, Lauren Zhang

**Affiliations:** U.S. Food and Drug Administration, Center for Food Safety and Applied Nutrition, Office of Regulatory Science, 5001 Campus Drive, College Park, MD 20740, USA; lauren.zhang@fda.hhs.gov

**Keywords:** patulin, LC-APCI-MS/MS, automated sample preparation

## Abstract

Patulin, a toxic mycotoxin, can contaminate apple-derived products. The FDA has established an action level of 50 ppb (ng/g) for patulin in apple juice and apple juice products. To effectively monitor this mycotoxin, there is a need for adequate analytical methods that can reliably and efficiently determine patulin levels. In this work, we developed an automated sample preparation workflow followed by liquid chromatography–atmospheric pressure chemical ionization tandem mass spectrometry (LC-APCI-MS/MS) detection to identify and quantify patulin in a single method, further expanding testing capabilities for monitoring patulin in foods compared to traditional optical methods. Using a robotic sample preparation system, apple juice, apple cider, apple puree, apple-based baby food, applesauce, fruit rolls, and fruit jam were fortified with ^13^C-patulin and extracted using dichloromethane (DCM) without human intervention, followed by an LC-APCI-MS/MS analysis in negative ionization mode. The method achieved a limit of quantification of 4.0 ng/g and linearity ranging from 2 to 1000 ng/mL (r^2^ > 0.99). Quantitation was performed with isotope dilution using ^13^C-patulin as an internal standard and solvent calibration standards. Average recoveries (relative standard deviations, RSD%) in seven spike matrices were 95% (9%) at 10 ng/g, 110% (5%) at 50 ng/g, 101% (7%) at 200 ng/g, and 104% (4%) at 1000 ng/g (*n* = 28). The ranges of within-matrix and between-matrix variability (RSD) were 3–8% and 4–9%, respectively. In incurred samples, the identity of patulin was further confirmed with a comparison of the information-dependent acquisition-enhanced product ion (IDA-EPI) MS/MS spectra to a reference standard. The metrological traceability of the patulin measurements in an incurred apple cider (21.1 ± 8.0 µg/g) and apple juice concentrate (56.6 ± 15.6 µg/g) was established using a certified reference material and calibration data to demonstrate data confidence intervals (k = 2, 95% confidence interval).

## 1. Introduction

Apple juice and apple-based foods are a convenient way for consumers, especially for children, to increase fruit consumption and improve overall diet quality. Apple juice consumption in the U.S. is estimated to be around 7.3 L per person annually, making it the second most consumed juice product [1,2]. However, apple harvest and storage practices may be susceptible to patulin, a toxic mycotoxin excreted by *Penicillium* and *Aspergillus* fungi (particularly *Penicillium expansum*), which can grow on fruits [3]. To reduce the risk of dietary exposure to patulin, the FDA has established an action level of 50 ppb for patulin in apple juice and apple juice products [4]. Currently, FDA field laboratories analyze compliance samples for patulin using liquid chromatography–UV [5], and in cases of non-compliance, the identity of patulin must be confirmed using mass spectrometry [6]. However, having two separate analyses that require two different analytical techniques is cumbersome. In addition, sample preparation has been largely manual, making patulin analyses labor intensive. 

To replace conventional manual sample preparation and achieve simultaneous identification and quantification of patulin in one analysis, an automated sample preparation procedure and liquid chromatography–tandem mass spectrometry (LC-MS/MS) platform was investigated. An automated sample preparation workflow, previously demonstrated for mycotoxin analyses [7], was optimized for patulin in different matrix types using integrated robotic tools to improve the efficiency of repetitive and high-volume tasks compared to manual operations. Sample preparation was paired with liquid chromatography–atmospheric pressure chemical ionization tandem mass spectrometry (LC-APCI-MS/MS), providing enhanced sensitivity and selectivity for patulin compared to optical methods. 

At the time of this study, most LC-MS-based methods for patulin analyses utilize electrospray ionization (ESI). Fewer studies have explored alternative ionization techniques, such as atmospheric pressure photo ionization (APPI) or atmospheric pressure chemical ionization (APCI), for the determination of patulin [8,9,10,11]. Dopant-assisted APPI enhances photo-ionization efficiency; however, it requires an evaluation of various dopant solvents, flow rates, and a solvent pump, adding complexity to any APPI-based method for a routine patulin analysis [10,11]. In contrast, APCI, similar to chemical ionization (CI) but performed at atmospheric pressure, can share the same general setup as ESI and be coupled with LC. In APCI, heat and a nebulization gas form an aerosol of the eluent from an LC system. Ions are formed in the gas phase using a corona discharge [12,13]. These features make APCI well-suited for ionizing low-mass and thermally stable compounds. Additionally, APCI is often less susceptible to matrix effects compared to ESI [14], offering an advantage to the measurement of patulin in a wide range of matrix sources.

In previous studies, we focused on matrix-induced signal suppression and enhancement—a distinctive technical challenge associated with LC-MS. Although using matrix-matched calibration standards [15,16] can reduce matrix effects on quantitation, stable isotope dilution is the preferred method for patulin quantitation [17,18]. The utilization of commercially available ^13^C-uniformly labeled mycotoxins has made stable isotope dilution a common practice within the agency, particularly for an LC-MS-based mycotoxin analysis. This widely accepted approach eliminates the need for matrix-matched calibration standards or standard addition and minimizes influences of sample preparation and matrix on quantitation with LC-MS. 

There are multiple sample preparation methods used for apple juice and apple-derived food samples analyzed with LC-MS, including direct extraction without cleanup, QuEChERS, derivatization, or liquid–liquid extraction followed by clean-up steps, such as solid-phase extraction (SPE) [19,20,21,22,23]. Clean-up steps can often include processes such as solvent exchange, evaporation, and reconstitution to enhance the compatibility of a given sample solvent with LC-MS or to improve sensitivity prior to submitting the extracts to an LC-MS analysis [24,25]. These sample preparation methods typically include extraction, shaking, centrifugation, liquid dispensing, filtration, capping/decapping, and SPE steps that all demand manual handling. Such labor-intensive tasks are not only time consuming but also systematically contribute to method variability (uncertainty).

**Figure 1:**
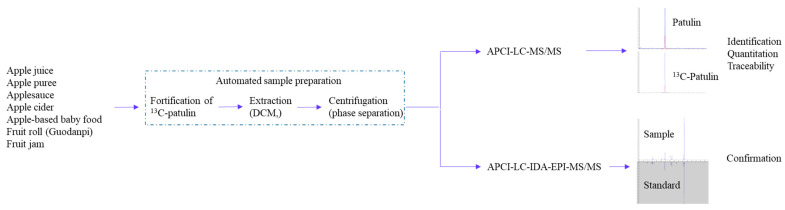
Automated sample preparation and LC-APCI-MS/MS for patulin analysis.

Varying degrees of laboratory automation have been demonstrated for mycotoxin analyses using LC-MS [26,27,28,29], although automated sample preparation has not been documented specifically for the analysis of patulin. Advances in automation technologies can facilitate the consolidation of various sample preparation tools onto one platform, potentially substituting laborious manual processes for a patulin analysis with completely automated sample preparation [7,30]. For our experiments, a robotic sample preparation system was selected, as this system is designed to seamlessly integrate and operate diverse sample preparation tools, executing predefined workflows. The primary advantage of such an integrated system lies in its ability to eliminate manual operations, particularly those occurring between and within different steps of sample preparation [7]. The automated sample preparation workflow was optimized, and a LC-APCI-MS/MS (Figure 1) was developed and validated for the determination of patulin in apple juice and apple-derived foods. The specific objectives were as follows:

- Develop a method for the identification and quantitation of patulin in apple juice and related foods following the FDA guidelines for the validation of chemical methods and mass spectrometry for a confirmation of identity [31,32].
- Develop an automated sample preparation workflow using a robotic sample preparation system to replace manual procedures and compare the method performance for each platform.
- Establish the metrological traceability of the patulin measurements generated by the automated sample preparation and LC-APCI-MS/MS method using certified reference materials (CRMs) as calibrants to provide systematic estimates of accuracy and uncertainty for data quality assessments [33].

## 2. Results and Discussion

The aim of this work was to develop an analytical method for the identification and quantitation of patulin using LC-MS and evaluate the use of an automated sample preparation workflow for the preparation of calibration standards and sample handling. LC-MS provides enhanced selectivity compared to optical methods while also reducing susceptibility to interference from co-eluted matrix components (e.g., 5-hydroxymethylfurfural) with similar UV absorbance [34]. By coupling LC-MS with stable isotope dilution, it is possible to perform direct extraction without the additional clean-up steps of conventional sample preparation processes, including solid phase extraction, evaporation, and reconstitution, which are inherently challenging for sample automation [35]. In the next sections, we will use performance data to demonstrate how a robotic sample preparation system coupled with LC-APCI-MS/MS may be applied to the identification and quantitation of patulin in samples of apple-derived products.

### 2.1. Comparison of APCI and ESI

**Figure 2:**
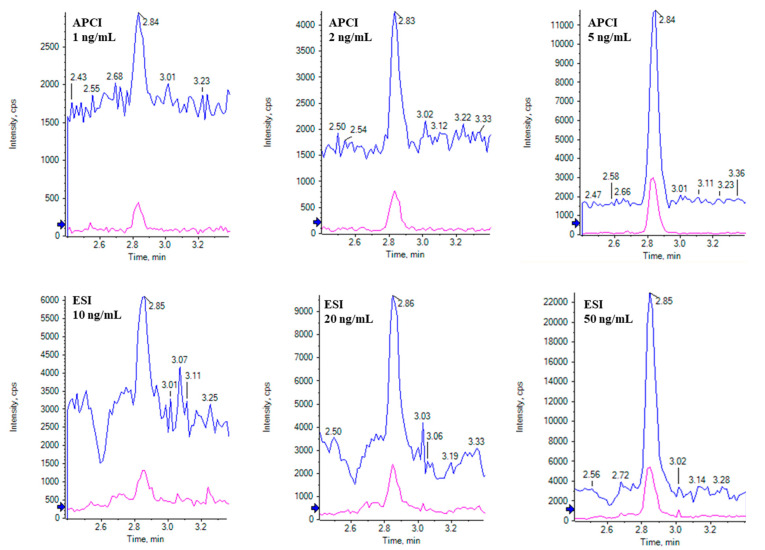
Comparison of patulin signal intensity under ESI and APCI using solvent standards.

Stable isotope dilution LC-MS has been proven to be the gold standard for quantitative analyses [36], offering simplified sample preparation and eliminating matrix-matched calibration standards for quantitation, but the success of this application relies on the sensitivity and specificity of LC-MS. Key components that would impact patulin sensitivity, including solvents, ionization mode, and polarity, were evaluated with APCI and ESI using optimized parameters for each mode. The signal intensity of patulin was directly influenced by the extent of ionization suppression during both ESI and APCI processes [37,38]. Figure 2 compares the signal response for patulin from APCI (1–5 ng/mL) and ESI (10–50 ng/mL) using solvent standards. For tested conditions, APCI demonstrated a superior ionization efficiency of patulin in both solvent and matrix conditions, exhibiting approximately 10 times higher sensitivity than ESI. To achieve a comparable performance, ESI would necessitate additional concentration steps, complicating sample preparation and reducing time efficiency. 

### 2.2. Comparison of Extraction Solvents

**Figure 3:**
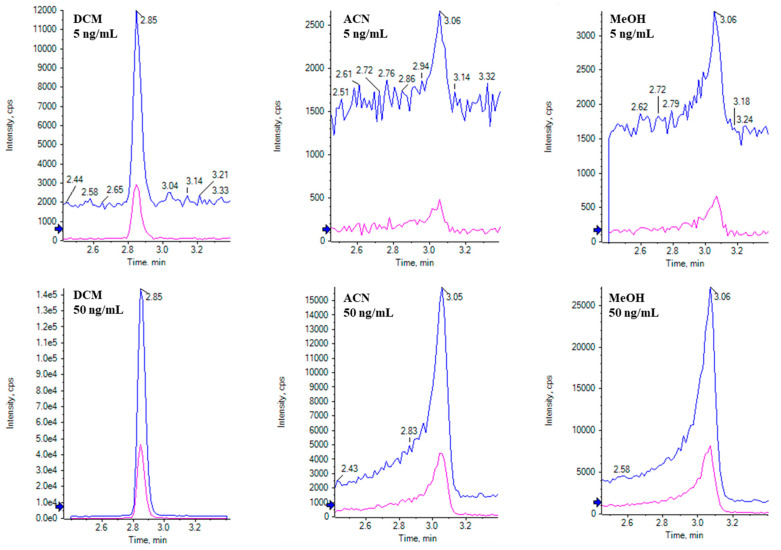
Comparison of acetonitrile, methanol, and dichloromethane using solvent standards.

To avoid the need for solvent exchanges to ensure sufficient chromatographic separation and symmetric peak shapes, extraction solvents that yield satisfactory extraction efficiency while being compatible with LC mobile phases are preferred. Although ethyl acetate, methanol, and acetonitrile are commonly used as extraction solvents for patulin analyses [7,39], DCM is a potential alternative for extracting moderate polar mycotoxins [40]. Ethyl acetate-based liquid–liquid extraction procedures may require multiple extractions and additional clean-up and concentration (e.g., SPE) [5,7,39], so it was not considered for this study. Methanol, acetonitrile, and DCM were compared in terms of patulin signal intensity and chromatography on our LC-MS instrument. Figure 3 clearly demonstrates that DCM outperformed the other two solvents with minimal peak tailing or fronting even when using an injection volume of 20 µL. Using DCM as the extraction solvent, extracts can be analyzed using LC-MS with dilution (10 times) at a target concentration of 50 ng/g, the FDA action level. Acetonitrile and methanol are less polar than the initial mobile phase conditions (95/5, water/methanol). This difference in polarity disrupts the distribution equilibrium between patulin and the stationary phase, resulting in inadequate retention on the column and the suboptimal chromatography of patulin at an injection volume of 20 μL. In contrast, DCM possesses higher polarity than the LC mobile phase. Consequently, patulin tends to concentrate at the column inlet, providing enhanced sensitivity at higher injection volumes (20 µL) without inducing peak broadening, as illustrated in Figure 3 [41].

### 2.3. Assessing the Robotic Sample Preparation System

The preparation of calibration standards requires frequent and consistently precise pipetting. We identified those repetitive activities as ones appropriate for automation and selected the Chemspeed Swing XL system to perform those specific tasks. The goal was to compare the performance of automated sample handling compared to manual preparations and evaluate its use for a sample analysis. Figure S1 illustrates the calibration standard preparation workflow, emphasizing crucial steps and tools. To demonstrate the consistency of the automated process, the workflow was employed to create eight batches of calibration standards, subsequently subjected to an LC-MS analysis. Each batch consisted of nine calibration standards ranging from 2 ng/mL to 1000 ng/mL. Table 1 summarizes the average and relative standard deviation (RSD) for each calibration concentration. Apart from the lowest calibration point at 2 ng/mL, exhibiting a 19% RSD, all other calibration points have an RSD ≤ 10%.

The sample preparation workflow (Figure S2) becomes more complex with additional tools and steps. Seven matrices underwent testing, wherein the Chemspeed system was programmed to execute spiking, liquid dispensing, extraction, centrifugation, capping, decapping, and the transportation of sample vials without human intervention. Each matrix was spiked at four concentrations (10, 50, 200, and 1000 ng/g) in quadruplicate preparations. An LC-APCI-MS/MS analysis was performed on the prepared extracts. Table 2 summarizes the recoveries and RSDs of the seven matrices. Recoveries range from 89–114%, with RSDs ranging from 0.4–11%. Within- and between-matrix variabilities were also calculated following ISO-5725-2 [42]. Within-matrix variabilities are 8% at 10 ng/g, 4% at 50 ng/g, 5% at 200 ng/g, and 3% at 1000 ng/g, while between-matrix variabilities are 9% at 10 ng/g, 5% at 50 ng/g, 7% at 200 ng/g, and 4% at 1000 ng/g. These results strongly indicate that the robotic sample preparation system coupled with LC-APCI-MS/MS can consistently conduct a quantitative analysis in representative food matrices suspected of patulin contamination.

For comparison, manual sample preparation was carried out across the seven matrices, and the results, including recoveries and relative standard deviations (RSDs), are listed in Table 3. Recoveries from the manual preparation vary between 85% and 125%, with RSDs ranging from 2% to 8%. Within-matrix variabilities are 6% for 10 ng/g, 4% for 50 ng/g, 4% for 200 ng/g, and 4% for 1000 ng/g, and between-matrix variabilities are 15% at 10 ng/g, 5% at 50 ng/g, 7% at 200 ng/g, and 6% at 1000 ng/g. The overlap of recoveries and RSDs between the automated workflow and manual procedure do not reveal any significant differences between the two sample preparation approaches, as illustrated in Figure S3 and confirmed by a box plot (Figure S4). No outliers (unusually large or small recoveries) were identified in the box plots for either procedure. A *t*-test verified that there is no statistical difference between the grand mean recoveries of the automated workflow (103%, *n* = 28) and the manual procedure (102%, *n* = 28). Evidently, both procedures yielded comparable results. 

A method limit of quantitation (LOQ) was estimated to be 4.0 ng/g using the lowest calibration point equivalent, 2.0 ng/mL and the dilution factor, 2. This limit was verified using the EPA protocol for the determination of the method detection limit [43]. Nine spike apple juice samples at 5.0 ng/g were prepared using the automated sample preparation workflow and analyzed with LC-APCI-MS/MS. The average and standard deviation (SD) of patulin in the nine spike samples was 4.62 ± 0.39 ng/g. The estimated method detection limit (MDL) = t_(8, 0.99)_ × SD = 2.896 × 0.39 ppb = 1.14 ng/g and LOQ = 3 × MDL = 3.42 ng/g. 

### 2.4. Identification, Confirmation, and Metrological Traceability of Patulin Measurements

**Figure 4:**
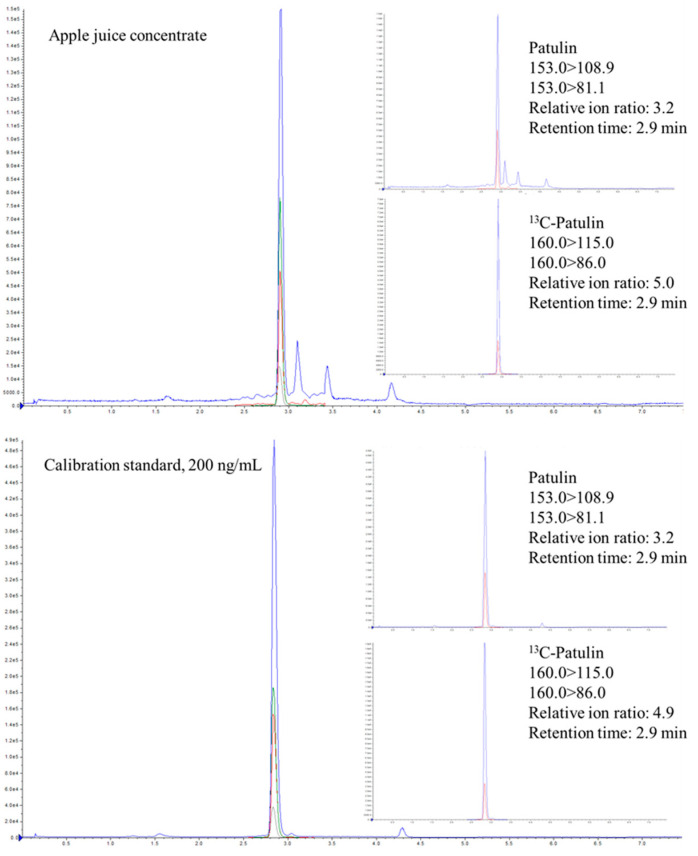
Identification of patulin in an incurred sample using LC-MS/MS.

**Figure 5:**
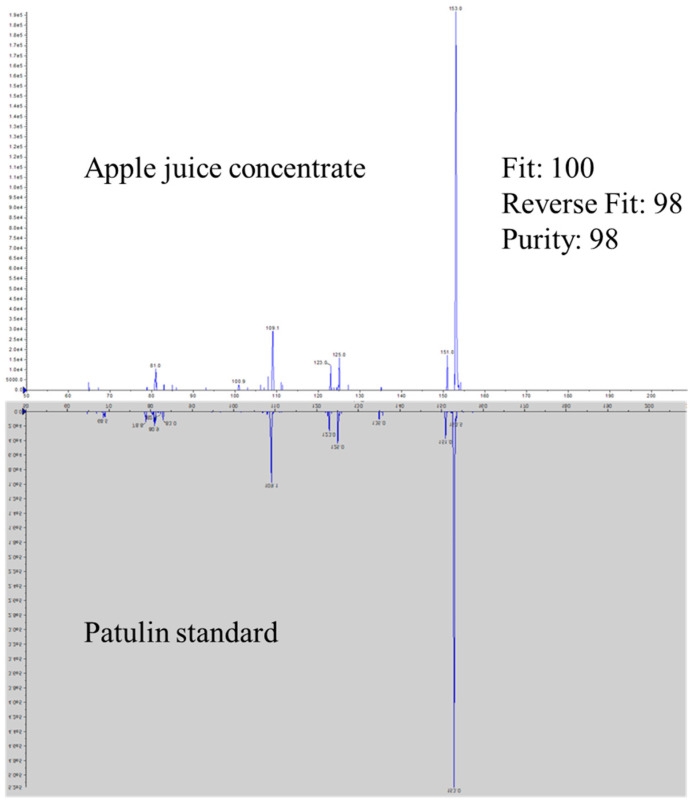
Confirmation of patulin using IDA-EPIMS/MS Spectra.

In total, sixteen apple juice, apple cider, and apple-derived food products were collected as a convenience sampling from local and online stores and analyzed using the automated sample preparation workflow and LC-APCI-MS/MS method. Patulin was detected in one apple cider and one apple juice concentrate. Identification was achieved following the identification criteria specified in the FDA Guidelines for the Validation of Chemical Methods for the FDA Foods Program, 3rd Edition (Figure 4) [31]. The identity of patulin was further confirmed using IDA-EPI spectra. The fit, reverse fit and purity were 100, 98, and 98, respectively (Figure 5), indicating a high degree of similarity between the spectra of detected patulin and reference spectra.

The significance of metrological traceability lies in its contribution to the accuracy, precision, and validity of the patulin measurements by relating them to internationally accepted references with certified purity and uncertainty. A lack of metrological traceability could compromise the reliability of the measurements of compliance samples, as it hinders a traceable estimation of the uncertainty associated with individual measurements.

Therefore, we determined the patulin concentrations for the two incurred samples prepared using the automated sample preparation workflow, along with their standard and expanded uncertainties (k = 2, approximately 95% confidence), using calibration data and established protocols [44,45]. A certified reference material (CRM) was utilized to estimate the uncertainty associated with LC-APCI-MS/MS, while the between-matrix variability (Table 2) was employed to estimate the uncertainty associated with sample preparation. Consequently, the patulin measurements of 21.1 ± 8.0 ng/g (k = 2) in apple cider and 56.6 ± 15.6 ng/g (k = 2) in apple juice concentrate can be traced metrologically to the International System of Units.

In the case of the two incurred samples generated using the manual preparation procedure, the respective patulin concentrations were 14.3 ± 11.4 ng/g and 52.6 ± 21.3 ng/g (k = 2, 95% confidence interval). The manual procedure yields wider confidence intervals for both measurements, and regardless of the sample preparation procedure, the confidence interval is wider at the lower concentration. The details of the statistical components used to estimate uncertainty are listed in Table S1. 

## 3. Conclusions

Considering the physicochemical properties of patulin, matrix components such as phenols, sugars, and pectin in apple juice and apple-derived food matrices could affect the recovery and identification of patulin. Conventionally, multiple extractions followed by solid-phase extraction (SPE) cleanup are conducted in an LC analysis to achieve satisfactory recovery rates and minimize the impact of matrix interferences. In an LC-MS analysis, matrix-matched calibration standards are necessary for quantitation if stable isotope dilution is not used. Additionally, automating the sample preparation using robotic devices is preferred to reduce manual operations for chemists. Therefore, this study demonstrates the development and validation of an LC-APCI-MS/MS method for patulin in apple juice and related products with automated sample preparation. This method provides a straightforward sample preparation process suitable for various apple-based food matrices, eliminating the need for matrix-specific methods. The performance of the method was assessed in terms of accuracy, precision, limits of detection, and uncertainty. Compared to manual procedures, the automated sample preparation workflow showed similar performance in the patulin analysis, replacing manual handling and improving efficiency and consistency in sample testing.

## 4. Experimental Section

### 4.1. Chemicals and Materials

HPLC grade acetonitrile, methanol, water, dichloromethane (ACS, 99.5%), MS grade formic acid, and ammonium formate were purchased from ThermoFisher Scientific (Waltham, MA, USA). A patulin certified reference material (CRM), GBW(E)100673, developed by the National Institute of Metrology of China, was purchased from A Chemtek Inc. (Waltham, MA, USA). The certified purity was 99.7% with an expanded uncertainty U (%) = 0.3 (k = 2). Apple juice, applesauce, apple-based baby food, apple puree, apple cider, fruit rolls, and fruit jam were purchased from local and online stores.

Stock solutions of patulin (100 μg/mL, in acetonitrile) and a stable isotope-labeled internal standard (IS), ^13^C-patulin (25 μg/mL, in acetonitrile), were purchased from Romer Laboratories, Inc. (Union, MO, USA). A working solution of ^13^C-patulin at 1000 µg/L was prepared in acetonitrile. Working solutions of patulin at 10, 20, 50, 100, 200, 500, 1000, 2000, 5000, and 10,000 µg/L (ppb) were prepared by diluting the stock solution using acetonitrile. All stock and working solutions were stored at −20 °C. Calibration standards (0.5 mL) at 1, 2, 5, 10, 20, 50, 100, 200, 500, and 1000 µg/L were prepared by diluting the corresponding working solutions 10 timesx. Each calibration standard (0.5 mL) was prepared by pipetting and mixing 400 µL of dichloromethane (DCM), 50 µL of a working solution, and 50 µL of the ^13^C-patulin working solution. The final concentration of ^13^C-patulin was 100 µg/L.

### 4.2. Robotic Sample Preparation System

A Chemspeed Swing XL system (Chemspeed Technologies, Füllinsdorf, Switzerland) was used to prepare calibration standards and samples throughout this study. Detailed hardware and software features of this Chemspeed system can be found in a previous study [7]. Briefly, the Swing XL system consisted of a platform (2.35 × 1.92 × 0.95 m, length × height × width), on which the following modular tools, racks, and plates were installed and used for the study: one overhead robotic arm with tool exchange interface, one DENSO robotic arm (DENSO Robotics), one four-channel liquid handling unit with a wash station, one gravimetric dispensing unit (GDU-v) for liquid transfers equipped with an analytical balance (gravimetric readability, 1 mg; volumetric accuracy: 1 µL), one gravimetric dispensing unit (GDU-p) for solid transfers equipped with an analytical balance (gravimetric readability, 1 mg), one Multigripper tool used for transport tasks, one Screw capper tool used to open and close vials with screw caps, one sample rack with 48 wells (6 × 8) for 2 mL LC autosampler vials, one shaking rack with 50 wells (5 × 10) for 15 mL centrifuge tubes, one shaking rack with 21 wells (3 × 7) for 50 mL centrifuge tubes, two cap plates (one for caps of 15 mL centrifuge tubes and the other for caps of 50 mL centrifuge tubes), and one syringe rack used to store 0.1 mL and 12.5 mL disposable syringes. A Sigma 4–16 KL centrifuge (Sigma, Osterode am Harz, Germany) was also attached to the platform. As directed by the AutoSuite localization and mapping features, the overhead robotic arm moved above the platform, picking up tools and completing various tasks. The DENSO robotic arm transferred samples to and from the centrifuge and the other devices used in the workflow. All Swing XL system actions were programmed using the Chemspeed AutoSuite software (version 2.4.20.1), which was also used to develop, simulate, and optimize workflows. These workflows will be described in plain language throughout the following sections.

### 4.3. Workflow for Preparing the Calibration Standards

Calibration standards were prepared by diluting working solutions and spiking them with ^13^C-patulin. Two solvent blanks (one spiked with ^13^C-patulin) and ten calibration standards ranging from 1 µg/L to 1000 µg/L were prepared with the Swing XL system as follows:

Step 1. Solvents, working solutions, and syringes were loaded. The positions of racks, plates, and tools were mapped by the software to ensure accurate pickup and replacement of items.

Step 2. The overhead robotic arm picked up the liquid dispensing unit (GDU-v) to load a 12.5 mL syringe from the syringe rack, aspirated 5 mL of DCM from a DCM solvent reservoir, and sequentially dispensed DCM into LC autosampler vials (400 µL each). The remaining DCM in the syringe was discarded into a waste container, after which the syringe was unloaded (placed back into the syringe rack).

Step 3. The GDU-v loaded a 0.1 mL syringe and moved it to the first working solution vial (10 µg/L). Using that syringe, the GDU-V aspirated 50 µL of air before drawing 50 µL of the working solution and then dispensing the solution into a 2 mL LC sample vial. To ensure the entire sample was ejected from the syringe, the dispensing volume was set at 100 µL. Then, the GDU-v repeated the above procedures to prepare the other calibration standards. The GDU-v unloaded the syringe back to the syringe rack after all the calibration solutions were prepared in the preloaded LC sample vials.

Step 4. The GDU-v loaded a 0.1 mL syringe and moved to the ^13^C-patulin stock solution vial. The syringe was programmed to aspirate 50 µL of air before drawing 50 µL of the ^13^C-patulin (IS) solution and then dispensing ^13^C-patulin solution into a 2 mL LC sample vial. The GDU-v unloaded the syringe back into the syringe rack after all the calibration standard vials were spiked with ^13^C-patulin. 

Step 5. This concluded the automated portion of the calibration standards. For these experiments, the Chemspeed had not been coupled with LC-MS instrument for online injection; therefore, the prepared calibration standards were manually transferred to LC-MS autosampler for LC-MS analysis.

### 4.4. Workflow for Sample Preparation

Samples were fortified with ^13^C-patulin and extracted. Automated samples were prepared using the Swing XL system as follows:

Step 1. The overhead robotic arm mounted the Screw capper and opened the 15 mL sample vials, which had been previously loaded onto the sample rack, which, in turn, was affixed to a shaker table. 

Step 2. The overhead robotic arm unmounted the Screw capper, mounted the GDU-v, loaded a 12.5 mL syringe, and dispensed liquid samples (e.g., apple juice, 1000 ± 25 mg each) into the 15 mL vials. Alternatively, homogenized solid samples (e.g., fruit rolls, 1000 ± 25 mg each) were dispersed using the GDU-p. Instead of loading a syringe, GDU-p loaded a dispensing container that was packed with homogenized fruit rolls.

Step 3. The 12.5 mL syringe or the dispensing container were unloaded, a 0.1 mL syringe was loaded, then 50 µL of ^13^C-IS were dispensed into each sample vial.

Step 4. The overhead arm mounted the GDU-v, mounted the 4-channel liquid handling unit, then added 2 mL of DCM to each sample vial.

Step 5. After unmounting the 4-channel liquid handling unit, the overhead arm mounted the Screw capper and capped the 15 mL sample vials. 

Step 6. The overhead arm unmounted the Screw capper, and the 15 mL sample vials were shaken at 1000 rpm for 3 min using the shaking feature of the sample rack. 

Step 7. The overhead arm mounted the Multigripper and transferred the 15 mL sample vials to the centrifuge buckets.

Step 8. The DENSO robotic arm moved the centrifuge buckets into the centrifuge and the human operator ensured the samples were centrifuged at 4500 rpm for 5 min.

Step 9. The DENSO robotic arm moved the centrifuge buckets out of the centrifuge, and the overhead arm used the Multigripper to move the sample vials back to the 15 mL sample rack.

Step 10. The overheard arm unmounted the Multigripper, mounted the Screw capper, opened the sample vials, and placed the caps on the cap plate.

Step 11. The overheard arm unmounted the Screw capper, mounted the 4-channel liquid handling unit, rinsed the tubing and needles, aspirated 0.5 mL of bottom layer (DCM), and transferred the extracts to 2 mL LC sample vials.

Step 12. The overheard arm rinsed and unmounted the 4-channel liquid handling unit.

Step 13. The overheard arm mounted the Screw capper and capped the sample vials.

Step 14. The overheard arm unmounted the Screw capper and returned to its home resting position.

Step 15. This concluded the automated portion of the sample preparation. Human operators then manually loaded the LC sample vials into the LC-MS autosampler for LC-MS analysis. 

Figures S1 and S2 are an illustration of the key tools and steps in the above workflows.

### 4.5. Manual Sample Preparation

Samples (1.00 ± 0.05 g) were weighed into 15 mL sample vials and spiked with 50 μL of ^13^C-patulin solution (1.0 ppm) followed by the addition of 2 mL of DCM. Using a Geno/Grinder, the sample vials were shaken for 3 min at 1000 rpm followed by centrifugation (5 min, 4200× *g*) to facilitate phase separation. Approximately 0.5 mL of the DCM extract was pipetted into an LC autosampler vial for LC-APCI-MS/MS analysis. Fruit rolls were processed using cryogenic milling. Samples were stored at −80 °C overnight and blended with dry ice using a Robot Coupe blender the next day, then stored in unsealed bags at −20 °C. After the CO_2_ sublimed, samples were extracted following the above procedures.

### 4.6. Recovery Studies

Recovery studies were conducted in apple juice, apple cider, apple puree, apple-based baby food, applesauce, fruit rolls, and fruit jam at 10, 50, 200, and 1000 µg/g. Blank samples (1.00 ± 0.05 g) were fortified using 100 µL of the working solutions at 100, 500, 2000, and 10,000 µg/L and 50 µL of the ^13^C-patulin working solution (1000 µg/L) and prepared as described above. Samples were prepared in quadruplicate at each fortification level. Patulin was quantitated using solvent calibration standards with the IS, ^13^C-patulin. Compound identification was based on retention time alignment within + 5% and the presence of two unique, structurally specific ions within a ±10% absolute unit ion ratio tolerance compared to a time-of-use standard [32].

### 4.7. LC-APCI-MS-MS Analysis

A Shimadzu Prominence/40 series (Columbia, MD, USA) LC was coupled with a SCIEX quadruple linear ion trap (QTRAP) 6500+ mass spectrometer with an APCI interface source (Foster City, CA, USA). An Agilent InfinityLab Poroshell 120 EC-C18 column (150 × 2.1 mm; 1.9 µm) and guard cartridge (5 × 2.1 mm; 2.7 µm) were used for LC separation (Santa Clara, CA, USA). The LC mobile phase consisted of 10 mM ammonium formate/0.1% formic acid/water (A) and 10 mM ammonium formate/0.1% formic acid/methanol (B). Gradient elution started at 5% B, ramped to 30% B in 1.5 min, and held for 2 min. The gradient was then ramped to 100% B in 0.5 min, returned to 5% B in 0.5 min, and re-equilibrated at 5% B for a total run time of 7.5 min at a flow rate of 0.3 mL/minute. The injection volume was 20 μL, and the column temperature was 40 °C. The mass spectrometer was operated in negative ionization mode with scheduled multiple reaction monitoring scanning (sMRM). Scan time was 1 s, and the sMRM detection window was 60 s. 

The optimized MRM transitions of patulin and ^13^C-patulin are listed in Table 4. APCI source-dependent parameters were set as follows: curtain gas, 36 au; nitrogen collision gas, high; source temperature, 600 °C; ion source gas 1, 60 psi; and nebulizer current, −3 µA. Identical LC conditions and MRM transitions of patulin were used for LC-ESI-MS/MS analysis. ESI ionization source parameters were set as follows: curtain gas, 36 psi; ion spray voltage, −4500 V; source temperature, 600 °C; and ion source gas 1 and gas 2, each at 60 psi.

### 4.8. LC-MS Information Dependent Analysis (IDA) and Enhanced Product Ion (EPI) Analysis

Confirmation of patulin identity was conducted following a previous IDA-EPI protocol [46]. Identical LC conditions were used for the LC-MS-IDA-EPI and LC-APCI-MS/MS analyses. Sciex OS 3.0, LibraryView, and Mycotoxin Library 1.0 (SCIEX, Framingham, MA, USA) were used to match reference EPI spectra and those collected from incurred samples. Collision gas (N_2_) pressure was set to “high.” The EPI spectra were collected within a range from *m*/*z* 50 to 200. The fill time of the ion trap was determined using dynamic fill time function. Product ions were scanned out of the QTRAP 6500+ at a rate of 10,000 amu/s. Source temperatures and voltages were the same as those used for the LC-APCI-MS/MS analysis. For the library searches, the mass tolerance window for precursor and fragment ions was set at 0.4 amu. The retention time window was 60 s, relative intensity threshold was 0.05, and the minimal purity was 50.

## Figures and Tables

**Table 1:** Average signal ratio of patulin to ^13^C-patulin and corresponding RSD (%) at different calibration points.

Conc. (ppb)	Average Signal Ratio of Patulin/^13^C-Patulin	RSD (%), *n* = 8 *
2	0.004	19
5	0.020	4
10	0.046	8
20	0.100	6
50	0.333	5
100	0.509	5
200	1.228	8
500	2.964	4
1000	6.415	4

* Eight batches of calibration standards ranging from 2–1000 ppb were prepared and analyzed on sperate days.

**Table 2:** Recoveries (RSDs, %, *n* = 4) of the within- and between-matrix variability of spike samples prepared using the automated sample preparation workflow.

Concentration (ng/g)	Spike Matrix							Range		Within-Matrix Variability (RSDr %)	Within-Matrix Variability (RSDR %)
	Apple Based Babyfood	Apple Juice	Apple Sauce	Apple Puree	Fruit Jam	Fruit Roll	Apple Cider	Recovery	RSD		
10	104 (4)	91 (6)	92 (8)	89 (7)	92 (8)	104 (9)	93 (11)	89–104	4–11	8	9
50	109 (3)	114 (7)	106 (2)	113 (4)	111 (5)	114 (4)	105 (4)	105–114	3–7	4	5
200	105 (1)	110 (4)	98 (0.4)	106 (6)	96 (3)	103 (4)	91 (7)	91–110	0.4–7	5	7
1000	104 (1)	107 (2)	96 (1)	106 (3)	104 (5)	107 (2)	104 (4)	96–107	1–5	3	4

**Table 3:** Recoveries (RSDs, %, *n* = 4) of the within- and between- matrix variability of spike samples prepared using the manual procedure.

Concentration (ng/g)	Spike Matrix							Range		Within-Matrix Variability (RSDr %)	Within-Matrix Variability (RSDR %)
	Apple Based Babyfood	Apple Juice	Apple Sauce	Apple Puree	Fruit Jam	Fruit Roll	Apple Cider	Recovery	RSD		
10	91 (8)	96 (5)	119 (7)	85 (7)	100 (7)	102 (2)	125 (6)	91–125	2–8	6	15
50	115 (5)	107 (5)	110 (4)	106 (2)	113 (5)	103 (2)	113 (1)	103–115	2–5	4	5
200	96 (5)	93 (2)	92 (3)	86 (2)	90 (3)	96 (4)	105 (4)	86–105	2–5	4	7
1000	109 (4)	103 (2)	100 (2)	96 (6)	104 (4)	95 (7)	106 (2)	95–109	2–7	4	6

**Table 4:** Patulin MRM transitions and compound-dependent parameters.

Analytes	Q1 Mass (Da)	Q3 Mass (Da)	Retention Time (min)	DP (eV)	EP (eV)	CE (eV)	CXP (eV)
Patulin	153.0	108.9	2.9	−5	−10	−12	−29
	153.0	81.1	2.9	−5	−10	−12	−5
^13^C-Patulin	160.0	115.0	2.9	−20	−10	−12	−11
	160.0	86.0	2.9	−20	−10	−12	−11

## Data Availability

Data available on request following Public Access to Results of FDA-Funded Scientific Research.

## Supplementary Materials

The following supporting information can be downloaded at: https://www.mdpi.com/article/10.3390/toxins16060238/s1, Figure S1, Chemspeed workflow for calibration standard preparation; Figure S2. Chemspeed workflow for automated sample preparation; Figure S3. Overlap of recoveries and RSDs of automated and manual sample preparation; Figure S4. Boxplot of recoveries (range, mean, and lower and upper quartiles) of automated and manual sample preparation. No outliers were detected; Table S1. Calibration standards, sample preparation, and associated statistics used for uncertainty estimation of patulin measurements in incurred samples; Supplemental Information: Metrological traceability of patulin measurements were established using a CRM and calibration data.

## Abbreviations

Chemical ionization	(CI)
Certified reference material	(CRM)
Dichloromethane	(DCM)
Electrospray ionization	(ESI)
Gravimetric dispensing unit	(GDU)
Information-dependent acquisition-enhanced product ion	(IDA-EPI)
Liquid chromatography–atmospheric pressure chemical ionization tandem mass spectrometry	(LC-APCI-MS/MS)
Limit of quantitation	(LOQ)
Method detection limit	(MDL)
Quadruple linear ion trap	(QTRAP)
Quick Easy Cheap Effective Rugged Safe	(QuEChERS)
Relative standard deviations	(RSD)
Scheduled multiple reaction monitoring scanning	(sMRM)
Solid-phase extraction	(SPE)
Standard deviation	(SD)
