# Supplementary material for: Determination of Patulin in Apple Juice and Apple-Derived Products Using a Robotic Sample Preparation System and LC-APCI-MS/MS
Source: Toxins (Basel). 2024 May 23;16(6):238. doi: 10.3390/toxins16060238 (PMC11209136; doi:10.3390/toxins16060238)
Supplement: Supplementary file 1 [file toxins-16-00238-s001.zip › toxins-3011193-supplementary.pdf]

Determination of Patulin in Apple Juice and Apple-Derived Products Using a Robotic Sample Preparation System and LC-APCI-MS/MS

Kai Zhang and Lauren Zhang

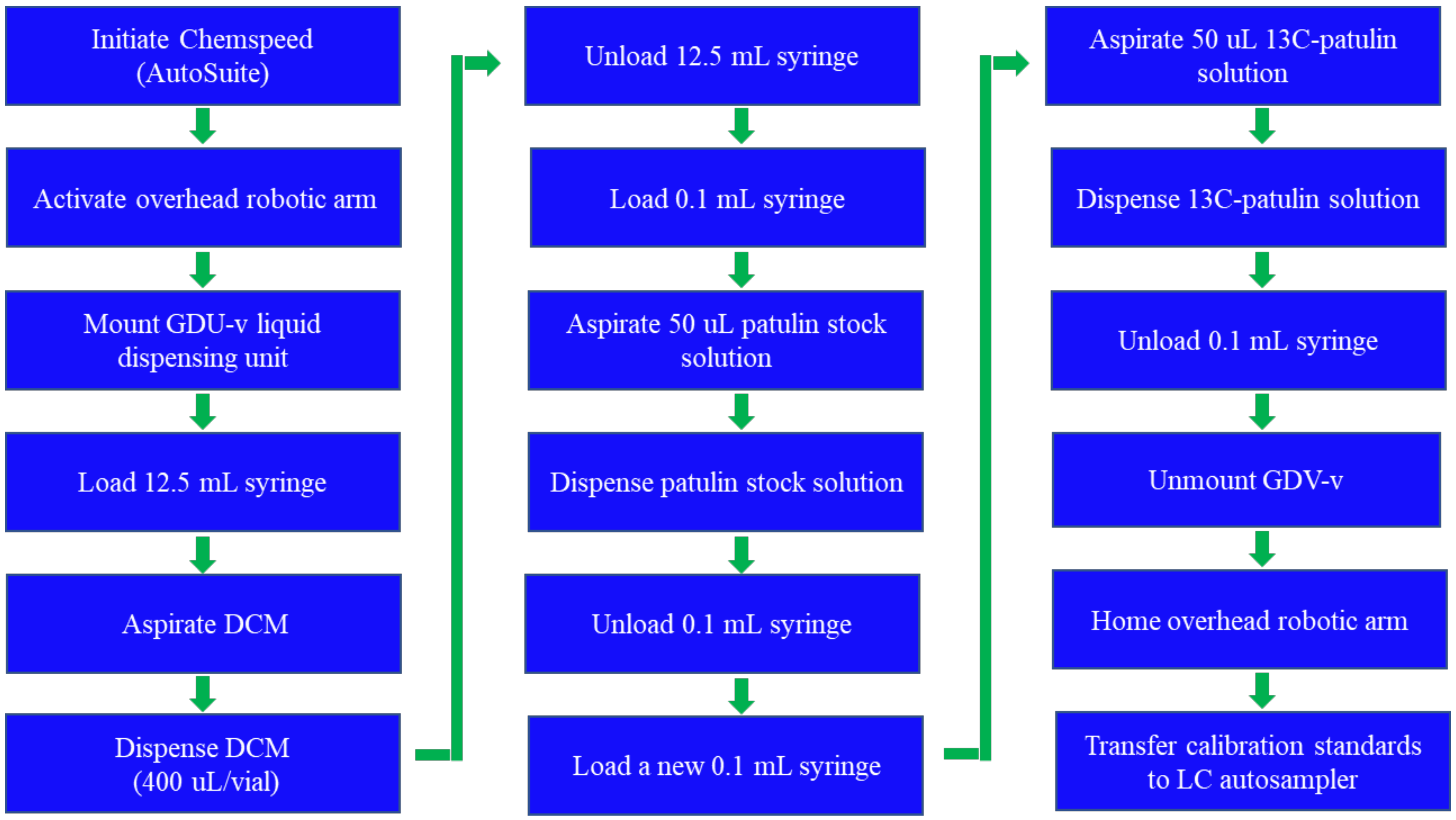

Figure S1. Chemspeed workflow for calibration standard preparation.

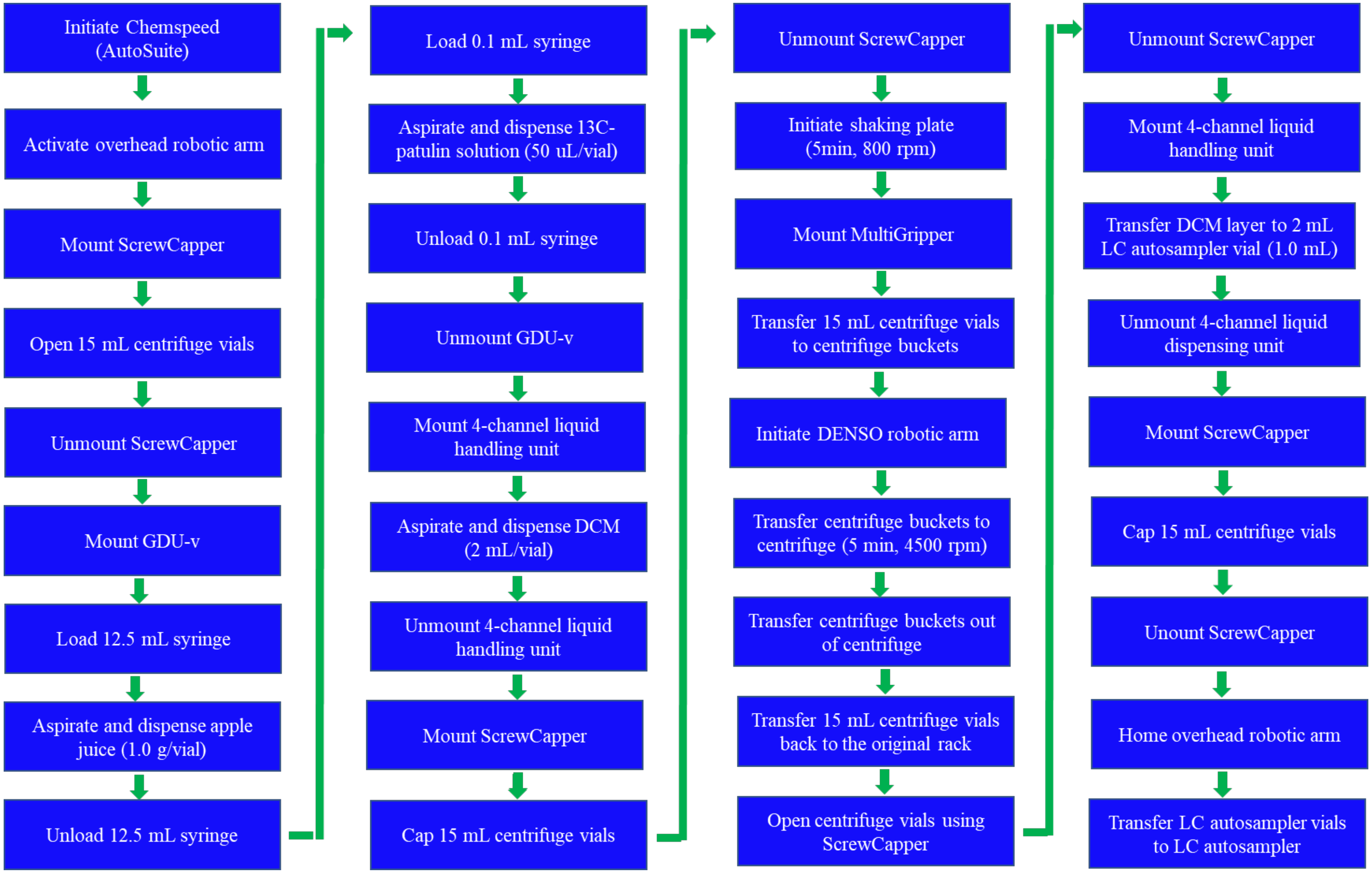

Figure S2. Chemspeed workflow for automated sample preparation.

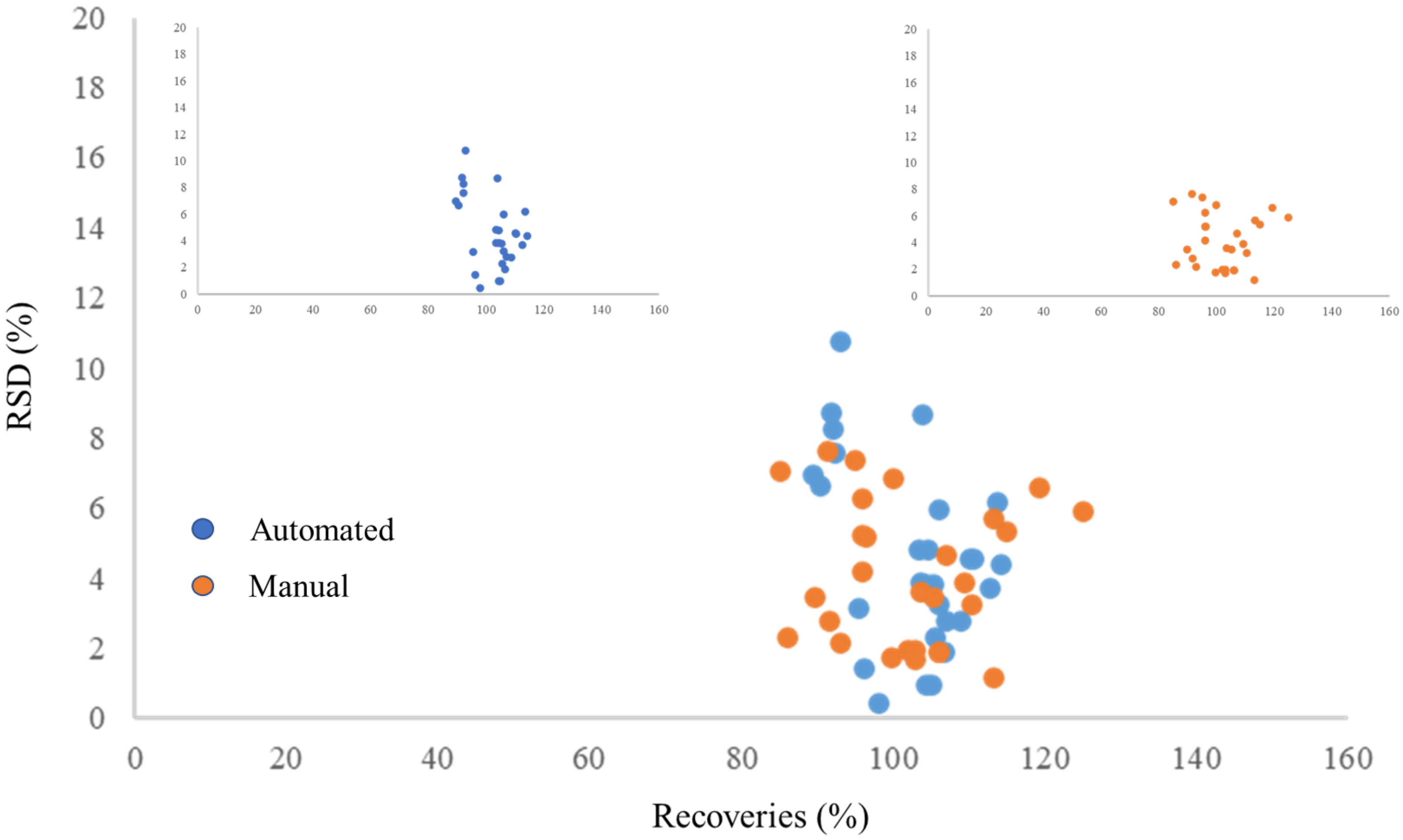

Figure S3. Overlap of recoveries and RSDs of automated and manual sample preparation.

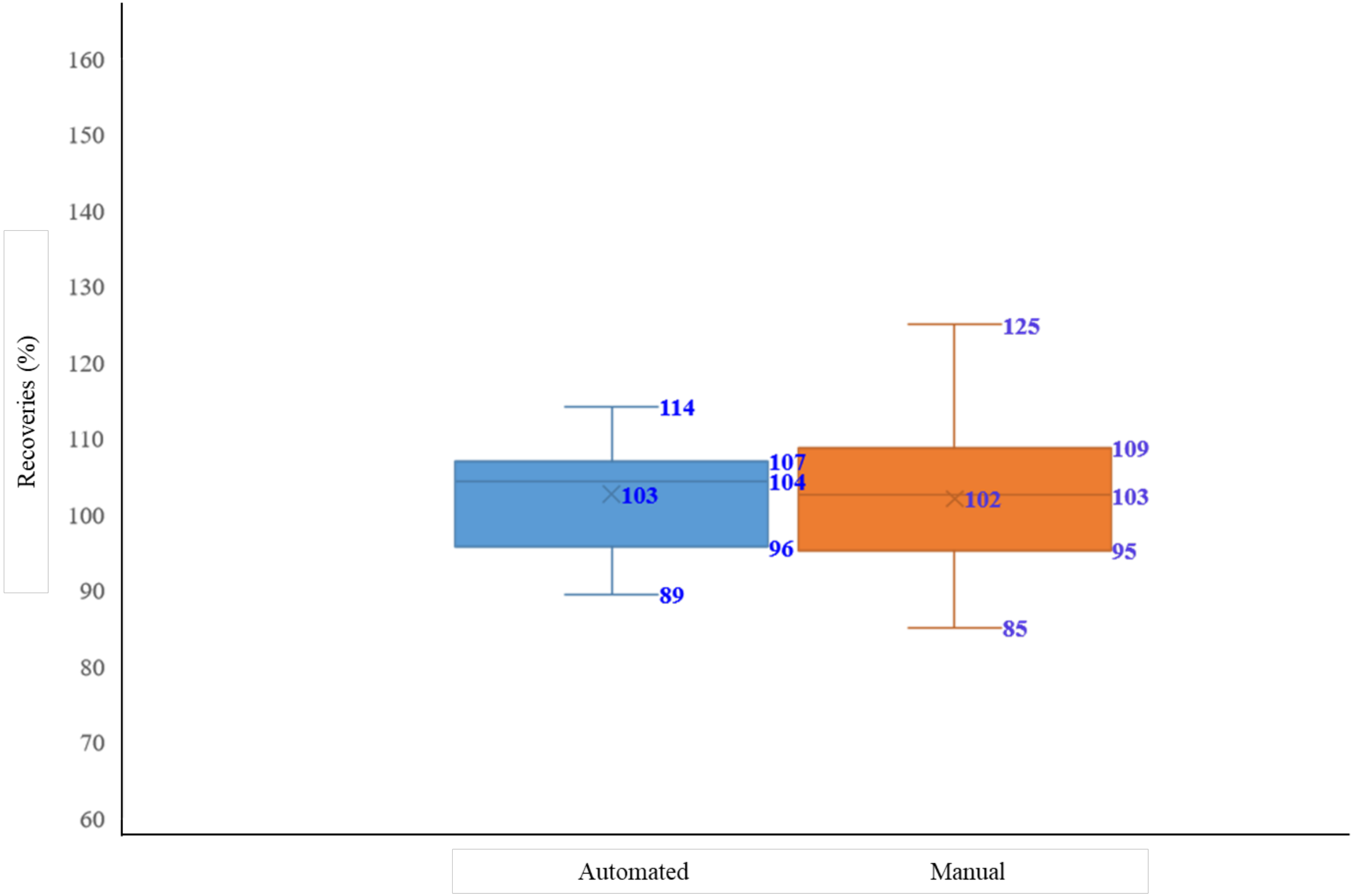

Figure S4. Boxplot of recoveries (range, mean, and lower and upper quartiles) of automated and manual sample preparation. No outliers were detected.

Table S1. Calibration standards, sample preparation, and associated statistics used for uncertainty estimation of patulin measurements in incurred samples.

Statistical components	Automated	Manual
Slope coefficient, a	0.006257	0.005724
Standard error of slope, S_a	0.000066	0.000107
Coefficient of determination, R^2	0.999211	0.997570
Intercept, b	-0.036147	-0.009426
Standard error in intercept, S_b	0.023154	0.037208
Average of calibration Conc., C_{cal}	196	196
Variance of calibration Conc., V_{cal}	747377	747377
Average of patulin/^{13}C-patulin ratio in calibration standards, Y_{cal}	1.190024	1.112320
Standard error in patulin/^{13}C-patulin ratio, S_{r}	0.057439	0.092301
Average of patulin/^{13}C-patulin ratio in sample, Y_{sample}	0.230336	0.172715
$Y = (Y_{\mathrm{sample}} - Y_{\mathrm{cal}})^2$	0.921002	0.882856
$X = \mathrm{a}^2\ V_{\mathrm{cal}}$	29.3	24.5
Average measured patulin in the sample (µg/g, n =4), C_{sample}	21.1	14.3
Standard error of C_{sample} based on a nine-point calibration curve (µg/g), S_{sample}	2.9	5.1
Relative standard uncertainty of patulin in the sample, u_{sample}	0.14	0.36
Relative standard uncertainty of patulin CRM, u_{CRM}	0.0015	0.0015
Relative standard uncertainty of LC-APCI-MS/MS, $u_{\mathrm{LC\text{-}MS}} = (u_{sample}^2 + u_{CRM}^2)^{0.5}$	0.14	0.36
Relative standard uncertainty of sample preparation ($u_{\mathrm{sample\ preparation}}$)	0.13	0.18
Relative standard uncertainty of balance, $u_{\mathrm{gravimetaric\ calibration}}$	0.001	0.000025
Relative standard uncertainty of pippetting, $u_{\mathrm{volumetric\ calibration}}$	0.01	0.006
Relative standard uncertainty, $u = (u_{\mathrm{LC\text{-}MS}}^2 + u_{\mathrm{sample\ preparation}}^2 + u_{\mathrm{gravimetaric\ calibration}}^2 + u_{\mathrm{volumetric\ calibration}}^2)^{0.5}$	0.19	0.40
Relative expanded uncertainty (k=2), $U = u \times \mathrm{k}$	0.38	0.80
Patulin concentration with expanded uncertainty in the sample (ng/g, k=2)	21.1 ± 8.0	14.3 ± 11.4

Supplemental Information

Metrological traceability of patulin measurements were established using a CRM and calibration data.

A bottom-up approach was used to identify key uncertainty sources. Standard (*u*) and expanded (*U*) uncertainty (k=2, 95% level of confidence) of the two patulin measurements were calculated using the relative standard uncertainty from major contributing factors, including an LC-MS instrument, sample preparation, the CRM, and gravimetric and volumetric apparatuses used for the analysis (JCGM 100:2008).

$$u = (\sum_{i=1}^{n} u_i^2)^{1/2} = (u_{\text{instrument}}^2 + u_{\text{sample preparation}}^2 + u_{\text{gravimetric}}^2 + u_{\text{volumetric}}^2)^{1/2}$$

$$u_{\text{instrument}} = (u^2_{\text{samp}} + u^2_{\text{CRM}})^{1/2} = 0.14$$

$$u_{\text{sample preparation}} = 0.13$$

The relative standard uncertainty of the method was calculated using pooled uncertainty data from the between-matrix variabilities at 10, 50, 200, and 1000 ppb (Table 3).

$$u_{\text{gravimetric}} = 0.001$$

The relative standard uncertainty of the analytical balance of GDU-v was estimated using the readability, 1 mg. The relative uncertainty for a 1,000 mg test portion is 0.001.

$$u_{\text{volumetric}} = 0.01$$

The relative standard uncertainty (1 μL) of the 100 μL pipettes was obtained from the calibration report. This yields a relative standard uncertainty of 0.01.

$$u = (u_{\text{instrument}}^2 + u_{\text{sample preparation}}^2 + u_{\text{gravimetric}}^2 + u_{\text{volumetric}}^2)^{1/2} = (0.13^2 + 0.14^2 + 0.001^2 + 0.01^2)^{1/2} = 0.19$$

$$U = k \times u \ (k = 2)$$

$$U = 2 \times 0.19 = 0.38 \ (\text{k}=2)$$
